# Impact of Cultural and Linguistic Maintenance on Mental Health Outcomes in Migrant Adolescents: Protocol for a Scoping Review

**DOI:** 10.2196/40143

**Published:** 2023-06-20

**Authors:** Anila Hasnain, John Hajek, Rohan Borschmann

**Affiliations:** 1 Research Unit for Multilingualism and Cross-Cultural Communication, School of Languages and Linguistics University of Melbourne Melbourne Australia; 2 Centre for Mental Health, Melbourne School of Population and Global Health University of Melbourne Melbourne Australia; 3 Department of Psychiatry Warneford Hospital University of Oxford Oxford United Kingdom; 4 Centre for Adolescent Health Murdoch Children's Research Institute Melbourne Australia; 5 Melbourne School of Psychological Sciences University of Melbourne Melbourne Australia; 6 School of Population Health Curtin University Perth Australia

**Keywords:** PRISMA, PRISMA-ScR, PRISMA-P, youth, risk factors, protective factors, suicide, depression, culture, language knowledge, acculturation, heritage

## Abstract

**Background:**

There is no consensus on how the disruption or maintenance of heritage culture and language affect mental health outcomes in adolescents with a migrant (also known as “immigrant” or “international migrant”) background. Even though previous literature reviews have investigated the association between acculturation and mental health in migrants, none have explicitly focused on adolescents.

**Objective:**

The aim of the scoping review described in this protocol is to understand (1) the focus, scope, and nature of quantitative empirical research investigating heritage cultural maintenance, including linguistic maintenance, and mental health outcomes in adolescents with a migrant background worldwide and (2) the potential effects of cultural and linguistic maintenance or disruption on migrant adolescent mental health outcomes.

**Methods:**

A total of 11 key electronic health, medical, social science, and language databases (APA PsycArticles Full Text; Embase Classic+Embase; Ovid MEDLINE All and Epub Ahead of Print, In-Process, In-Data-Review and Other Non-Indexed Citations and Daily; Ovid MEDLINE All; APA PsycInfo; University of Melbourne full-text journals; Science Citation Index Expanded; Social Sciences Citation Index; Arts & Humanities Citation Index; Scopus; Linguistics and Language Behavior Abstracts) were searched. Databases were searched without time restrictions from the beginning of their coverage. Publication date, location, and quantitative study design (except for literature reviews) were not restricted; however, the search was only conducted in English. Data from included studies will be extracted using a template with predefined data items, and results will be summarized in a structured, narrative summary.

**Results:**

A search was conducted on April 20, 2021, returning 2569 results. We are currently at the final stages of screening titles and abstracts of our search results, which will be followed by a full-text review and the data extraction of included studies. We expect to submit the full review for publication by the end of 2023.

**Conclusions:**

The scoping review aims to provide a better understanding of existing research on the association between cultural (including linguistic) maintenance and mental health in migrant adolescents. It will help identify gaps in the existing literature and develop hypotheses that could inform future research, eventually facilitating the development of targeted prevention initiatives and improving migrant adolescents’ well-being.

**International Registered Report Identifier (IRRID):**

DERR1-10.2196/40143

## Introduction

### Background

The World Migration Report 2022 stated that 1 in 30 people globally resides in a country that differs from their birth country and that this number has increased over the last 5 decades [1]. With most migration streams flowing from low-income to high-income countries [1], many migrants experience differences between their heritage and host cultures.

Previous research into acculturation (ie, the impact of interacting with other cultures on an individual) has highlighted 2 key issues. First, the issue of cultural maintenance, which is the extent to which heritage cultural identity is considered important and practices are continued. Second, the issue of deciding on the extent of contact and participation (ie, interacting with the host culture or remaining within heritage cultural groups) [2]. Based on these 2 issues, Berry and Sam [2] proposed four acculturation strategies: (1) integration (whereby heritage culture is maintained while seeking to interact with other groups), (2) assimilation (whereby heritage culture is not maintained while interactions with other groups are sought), (3) separation (whereby heritage culture is maintained while wishing to avoid interactions with other groups), and (4) marginalization (whereby cultural maintenance is perceived as difficult, and little interest is demonstrated in interacting with other groups).

A considerable amount of research has examined the association between acculturation and mental health outcomes, including several systematic reviews [3,4] and meta-analyses [5,6]. However, despite age being identified as a key component of the association between acculturation and mental health [6], no review to date has examined how cultural maintenance affects mental health outcomes in migrant adolescents (aged 10-24 years [7]).

Marked as the transition into adult life, adolescence is a life phase of great change. Adolescence has been identified as “a second sensitive developmental period in which puberty and rapid brain maturation lead to new sets of behaviours and capacities that trigger or enable transitions in family, peer, and educational domains, and in health behaviours” [8]. As these transitions affect a child’s future health outcomes [8], understanding factors shaping adolescents’ mental health outcomes is important to support their healthy development.

Even though Viner and colleagues [8] caution that their ecological analysis does not imply causality, they found that “national wealth, inequality, and education had the largest effect sizes...and were associated with the largest range of health outcomes in young people.” They further identified that similar factors, related to family, peers, and education, can help protect adolescents’ health across countries [8].

Adolescents with a migrant background—specifically, first- or second-generation migrants—are faced with the added complexity of navigating this intense transitional phase within a multicultural or multilingual context. In fact, some research has identified a migrant background to be a predictor of poor mental health outcomes in adolescents [9]. A study in a regional child and adolescent mental health service in Australia reported that the majority of mental health support was needed by Aboriginal youth, followed by Asian-European heritage youth, with the researchers pointing at the role loss and disconnection from heritage culture may play in poor mental health outcomes [10]. Hallett and colleagues [11] had even more poignant findings related to Indigenous cohorts, identifying native language knowledge as correlating with a reduction in Indigenous youth suicide in Indigenous communities in Canada. The extent to which a similar effect of language or culture maintenance on mental health outcomes may be evident among adolescents with a migrant background remains unknown.

There is a large body of adolescent mental health research studying the effectiveness of interventions [12], help-seeking behaviors [13,14], and the applicability of information technologies [15]. Müller and colleagues [16] conducted a scoping review on the effect bilingualism has on family and child well-being, which provides valuable insights. However, rather than specifically looking at bilingualism, we are interested in research investigating the association between heritage culture or language knowledge and mental health outcomes in migrant adolescents.

This scoping review will provide a better understanding of migrant adolescents’ ecological systems and identify modifiable risk factors associated with optimal mental health outcomes. This knowledge will inform future research leading to improved interventions and policies that contribute to improved mental health outcomes in migrant adolescents.

### Objectives

This protocol outlines the methodology of a scoping review that will address the following questions:

(1) What is the focus, scope, and nature of quantitative empirical research investigating heritage cultural maintenance, including language maintenance, and mental health outcomes in adolescents with a migrant background worldwide?

(2) What effects, if any, do cultural and linguistic maintenance or disruption have on migrant adolescent mental health outcomes?

## Methods

### Protocol Development

This protocol adheres to relevant sections of the Preferred Reporting Items for Systematic Review and Meta-Analyses–Protocols (PRISMA-P) [17], which we provide in the form of a checklist [18] in Multimedia Appendix 1.

### Scoping Review Methodology and Reporting

We conduct the scoping review following the methodological framework outlined by the Joanna Briggs Institute [19], which is based on the Arksey and O’Malley framework [20] and Levac and colleagues’ [21] elaborations. Reporting will follow the PRISMA Extension for Scoping Reviews (PRISMA-ScR) [22].

For an overview of the scoping review’s inclusion and exclusion criteria, please refer to Textbox 1.

**A summary of inclusion and exclusion criteria.**

**Inclusion Criteria**

- Published, peer-reviewed empirical studies.
- Any language (however, the search was only conducted in English, which indirectly excluded all studies without an English title, abstract, or keywords).
- Any publication date.
- Any study location.
- Adolescent study participants between 10 and 24 years.
- Studies focused on mental health outcomes, symptom severity, and diagnosis.
- Studies measuring mental health outcomes, specifically depression, anxiety, self-harm, suicide, substance misuse, and schizophrenia.
- Studies measuring any nondominant heritage culture or language.

**Exclusion Criteria**

- Qualitative studies, literature reviews, book chapters, dissertations, conference papers, conference abstracts, and editorials.
- Participants younger than 10 years, or those who are 25 years or older.
- Studies focused on help-seeking, or interventions in mental health dimension.
- Studies measuring trauma, self-esteem, life satisfaction, or well-being.
- Studies measuring the uptake of host language or culture without considering heritage language and cultural maintenance; studies measuring ethnic identity.

### Eligibility Criteria

#### Participants

We include empirical studies examining the effect of cultural maintenance, including linguistic maintenance, on mental health outcomes in migrant adolescents. Participant age is limited to 10-24 years, in line with the contemporary definition of adolescence [7].

#### Outcome Measures

The scoping review is interdisciplinary in nature and included studies must measure mental health and cultural or linguistic maintenance outcomes. Appropriate mental health outcomes include depression, anxiety, self-harm and suicide, substance misuse, and schizophrenia. Studies measuring only trauma, self-esteem, life satisfaction, or well-being are excluded. Studies measuring mental health outcomes without measuring culture or language maintenance are also excluded. Studies examining help-seeking or interventions are also not included.

Heritage language or cultural maintenance must be measured for studies to be included. We do not include studies that examine only the uptake of the host language or culture without examining heritage culture or language maintenance, but we consider findings related to host cultural orientation if heritage cultural orientation is measured.

#### Study Design

We include published, peer-reviewed empirical studies measuring cultural or linguistic maintenance and mental health outcomes in migrant adolescents. We exclude qualitative studies, book chapters, dissertations, conference papers, conference abstracts, and editorials. We do not include literature reviews as they may include studies that do not meet our inclusion criteria. However, we will screen and review the reference lists of relevant literature reviews to identify studies not identified during our searches.

### Information Sources

A total of 11 electronic databases were searched on April 20, 2021, from the beginning of each database.

The electronic databases are APA PsycArticles Full Text (coverage from 1894); Embase Classic+Embase (from 1947); Ovid MEDLINE All and Epub Ahead of Print, In-Process, In-Data-Review and Other Non-Indexed Citations and Daily (from 1946); Ovid MEDLINE All (from 1946); APA PsycInfo (from 1806); University of Melbourne full-text journals; Science Citation Index Expanded (from 1900); Social Sciences Citation Index (from 1900); Arts & Humanities Citation Index (from 1975); Scopus (from 1970); Linguistics and Language Behavior Abstracts (from 1973).

We will also screen reference lists to identify relevant studies that the electronic search might not have identified.

### Search Strategy

The search strategy was shared with a university librarian, who provided feedback to further tailor the search strategy to each database. After the search of the electronic databases, the search results’ titles and abstracts are screened in a Microsoft Excel (Microsoft Corp) spreadsheet, applying the inclusion and exclusion criteria as outlined by this protocol. To be eligible for inclusion, studies must fulfill all 4 categories (coded in the search query with “AND”). Within each category, studies must meet at least 1 search term (coded in the search query with “OR”). The search query syntax was adjusted to meet specific database requirements. An example of the search command used for Ovid MEDLINE All is provided in Table 1.

**Table 1:** Ovid MEDLINE All search strategy.

Category	Operators	Search field	Fields
1. Linguistic and cultural component	N/A^a^	“language maintenance” OR “language knowledge” OR “language transmission” OR “cultural maintenance” OR “cultural knowledge” OR “cultural transmission”	All fields
2. Mental health outcome	AND	“mental health” OR suicid* OR depress* OR anxiet* OR anxious* OR self-injur* OR self-harm OR nssi OR “non-suicidal self-injury” OR “substance abuse” OR “drug abuse” OR “substance use” OR “substance misuse” OR “substance-related disorder” OR “substance use disorder” OR “SUD” OR resilien* OR schiz* OR vulnerab*	All fields
3. Age group	AND	adolescen* OR “young person” OR teen*	All fields
4. Immigrant background	AND	refugee* OR cald OR “culturally and linguistically diverse” OR immigrant OR migrant OR aborigin* OR indigenous OR “Torres Strait”	All fields

^a^N/A: not applicable.

### Study Records

#### Data Management and Selection Process

AH exported and consolidated all electronic database study results’ titles and abstracts into a single Microsoft Excel spreadsheet before removing duplicates. AH is currently finalizing the screening of titles and abstracts, applying the inclusion and exclusion criteria, and recording reasons for inclusion or exclusion. JH and RB are involved in the screening of search results, checking 20% of included and excluded studies to test the validity of the approach and discuss potential alterations.

In the next stage, AH will download the full text of studies whose title and abstract appear relevant or unclear in answering the research question into citation management software Zotero (Corporation for Digital Scholarship). Each study will be read in full and assessed against each inclusion criterion using a simple charting form covering each of the 4 search categories. AH will consult the appropriate expert in the team to discuss studies that she is unsure about. If they cannot agree, the third team member will decide. JH and RB will review all included studies and at least 20% of excluded studies to detect and correct potential errors.

#### Data Collection Process and Data Items

AH will collect data using 2 separate spreadsheets. In the first spreadsheet, AH will record extracted information for each included study: authors, year, final sample size, other included cohorts (eg, parents), participant information (age, heritage, and culture), and geographical location.

In the second spreadsheet, AH will code data in a binary table, which will allow sorting and comparing study characteristics. Expected information categories that will be coded at the top level will be related to frameworks, the terminology used, cultural or linguistic measures, mental health outcomes measured, scales used, as well as key findings about this paper’s second research question. These findings will be shared, reviewed, and discussed with JH and RB.

### Outcomes and Prioritization

The primary focus of this review is to understand the research investigating the association between cultural or linguistic maintenance and mental health outcomes in migrant adolescents. As we are investigating a multidisciplinary field of study, there is a possibility of studies having heterogeneous research perspectives and findings. We aim to be rigorous in including only studies that fulfill all 4 search categories. When studies meet criteria in all 4 categories, we will also report on findings that may directly or indirectly affect the association between cultural maintenance and mental health outcomes, such as the effect of host cultural orientation, as well as moderating or mediating factors. This will allow us to understand the identified phenomena better.

### Data Synthesis

We will provide a descriptive overview of included studies, stating sample size, age, and location, as well as mental health outcomes and cultural or linguistic maintenance factors measured. We will then provide a structured, narrative summary of study findings about our second research question in text and—depending on feasibility—table form. We will report commonalities and differences in findings, explore potential hypotheses, and identify gaps in the research. We will not assess the quality or bias of studies, grade evidence, or include a quantitative synthesis of evidence.

### Ethical Considerations

Our review does not require ethical approval, as it reports on findings from published studies that have already obtained ethics approval. We will disseminate the findings of the scoping review through a peer-reviewed journal article and national and international conference presentations.

## Results

Applying the methodology outlined in this protocol, we conducted a search in April 2021, which returned 2569 results. We are in the process of screening titles and abstracts of these results. We expect to submit the scoping review for publication by the end of 2023.

## Discussion

### Overview

This scoping review protocol outlines a rigorous and replicable methodology for the review of empirical research investigating cultural maintenance, including language maintenance, and migrant adolescent mental health outcomes. To our knowledge, no comparable study has been conducted. The search conducted in April 2021 returned 2569 results.

We will report results in the scoping review following the PRISMA-ScR. Our search strategy included 11 relevant academic databases, as well as a grey literature search. We are screening titles and abstracts and will extract data from included studies using predefined charting items. This will allow us to compare the nature of study elements and identify trends and gaps in the existing literature.

We will also extract relevant findings to address our second research question and present the results in a narrative summary, which will possibly result in the suggestion of hypotheses or research questions that could inform future research or systematic reviews. The preliminary review of literature has shown that rather than inferring causality, it is important to interpret findings considering adolescents’ context. Some studies use an ecological approach [23] and, depending on our results, we are planning to use a similar approach to contextualize results.

Some existing reviews that were not adolescent-specific identified biculturalism to be advantageous on adjustment [5], and child well-being benefiting from bilingualism at home [16]. Our study will contribute to the existing literature, providing a better understanding of language or cultural maintenance, while specifically focusing on mental health outcomes in migrant adolescents who are and will constitute large cohorts within society.

Even though we conducted the search worldwide, we used only English search terms, limiting our results to studies with English titles, abstracts, and keywords. Included results that were written in another language are reviewed by a researcher proficient in the language. Another limitation of this study is that evidence will not be quantitatively synthesized in this review, as we will not do a quality or bias assessment of studies. Moreover, this highly multidisciplinary field of study leads to heterogeneous results from various disciplines (ie, psychology, adolescence research, and linguistics), which slows down the review process.

### Conclusion

The scoping review outlined by this protocol will provide a more cohesive understanding of existing multidisciplinary research into cultural and linguistic maintenance and mental health outcomes in migrant adolescents. This will provide a more comprehensive understanding of how youth mental health outcomes and cultural factors, including linguistic factors, have been investigated to date, and what future investigations may be required. The outlined scoping review therefore plays an important part in improving migrant adolescents’ mental health outcomes.

## Appendix

Multimedia Appendix 1 PRISMA-P checklist.

## Abbreviations

PRISMA-P: Preferred Reporting Items for Systematic Review and Meta-Analyses Protocols
PRISMA-ScR: Preferred Reporting Items for Systematic Review and Meta-Analyses extension for Scoping Reviews

## Data Availability

All data generated or analyzed during this study will be included in the published article (and its supplementary information files).
